# Simultaneous Electrochemical Detection of Dopamine and Ascorbic Acid Using an Iron Oxide/Reduced Graphene Oxide Modified Glassy Carbon Electrode

**DOI:** 10.3390/s140815227

**Published:** 2014-08-19

**Authors:** Teo Peik-See, Alagarsamy Pandikumar, Huang Nay-Ming, Lim Hong-Ngee, Yusran Sulaiman

**Affiliations:** 1 Low Dimensional Materials Research Centre, Department of Physics, Faculty of Science, University of Malaya, Kuala Lumpur 50603, Malaysia; E-Mail: pepsi88@hotmail.com; 2 Department of Chemistry, Faculty of Science, Universiti Putra Malaysia, UPM Serdang, Selangor 43400, Malaysia; E-Mails: janet_limhn@yahoo.com (L.H.-N.); yusran@upm.edu.my (Y.S.)

**Keywords:** graphene, magnetite, dopamine, ascorbic acid, biosensor, electrochemical sensor

## Abstract

The fabrication of an electrochemical sensor based on an iron oxide/graphene modified glassy carbon electrode (Fe_3_O_4_/rGO/GCE) and its simultaneous detection of dopamine (DA) and ascorbic acid (AA) is described here. The Fe_3_O_4_/rGO nanocomposite was synthesized *via* a simple, one step *in-situ* wet chemical method and characterized by different techniques. The presence of Fe_3_O_4_ nanoparticles on the surface of rGO sheets was confirmed by FESEM and TEM images. The electrochemical behavior of Fe_3_O_4_/rGO/GCE towards electrocatalytic oxidation of DA was investigated by cyclic voltammetry (CV) and differential pulse voltammetry (DPV) analysis. The electrochemical studies revealed that the Fe_3_O_4_/rGO/GCE dramatically increased the current response against the DA, due to the synergistic effect emerged between Fe_3_O_4_ and rGO. This implies that Fe_3_O_4_/rGO/GCE could exhibit excellent electrocatalytic activity and remarkable electron transfer kinetics towards the oxidation of DA. Moreover, the modified sensor electrode portrayed sensitivity and selectivity for simultaneous determination of AA and DA. The observed DPVs response linearly depends on AA and DA concentration in the range of 1–9 mM and 0.5–100 μM, with correlation coefficients of 0.995 and 0.996, respectively. The detection limit of (S/N = 3) was found to be 0.42 and 0.12 μM for AA and DA, respectively.

## 1. Introduction

Dopamine (DA) is one of the most important catecholamine neurotransmitters which are produced in the adrenal glands and several area of the brain. Moreover, DA is found to be the most abundant catecholamine that belongs to the family of inhibitory neurotransmitters involved in the central nervous, renal, hormonal and cardiovascular systems. Low levels or abnormalities in the DA concentration may lead to several neurological diseases, such as schizophrenia, Parkinson's disease [1], attention deficit hyperactivity disorder (ADHD) [2], restless legs syndrome (RLS) [3] and drug addiction. Owing to the strong electrochemical activity of DA, the determination of DA at sub-micro molar concentration level has gained immense attention among numerous researchers. Several methods have been developed for DA detection, including high performance liquid chromatography [4], chemiluminescence [5] and gas chromatography-mass spectrometry [6]. However, these analytical techniques have some limitations, such as being time-consuming, low-sensitivity and requiring expensive equipment, although they represent effective strategies for the detection of DA. Electrochemical sensors are a preferred alternative method because of their simple operation, fast response, time savings, low-cost, high-sensitivity, excellent selectivity and real-time detection. However, the electrochemical determination of DA is hindered by the coexistence of interfering compounds, such as ascorbic acid (AA) and uric acid (UA) in neural biological environment. Ascorbic acid, another electroactive species that plays an important role as an antioxidant in human metabolism, is the main interfering species that coexists with DA in the central nervous system. Through the electrochemical method, it is difficult to selectively sense the DA in the presence of high concentration levels of AA, because these two species are sharing nearly the same oxidation potential on the bare electrode, which results rather poor selectivity and sensitivity of DA detection [7].

In the traditional electrochemical sensor method, direct determination of DA is rare and causes poor DA response. Thus, the chemical modification of electrode surfaces has been developed to enhance the sensitivity and selectivity of electrochemical sensors. In recent years, sensing materials with high stability, good catalytic and excellent conductivity, such as polymers [8], carbonaceous materials [9] and metal oxide nanoparticle-based [10] nanocomposites have been successfully reported for the modification of bare GC electrode surfaces. Recently, carbonaceous material-modified electrode based electrochemical sensors have received widespread attention. Among them, graphene is extensively used to detect a wide range of analytes. Graphene is a two-dimensional (2D) one atom thick single layer of sp^2^-bonded carbon atoms densely packed in a honeycomb lattice [11] and it possesses high specific surface area, excellent thermal conductivity, extraordinary electrocatalytic activity and high charge mobility. Hence, the graphene-based nanocomposites hold great promise as ideal candidate sensing platforms for the development of electrochemical sensors and biosensors [12]. Magnetite (Fe_3_O_4_) nanoparticles with amazing properties *viz.* biocompatible, strong super-paramagnetic nature, low-toxicity and easy separation have gained much attention as catalysts in the fields of biomedical, biotechnological and sensing [13–18]. However, pure magnetite nanoparticles are chemically unstable and easily oxidize and hence they hinder their special properties in applications. Thus, incorporation of magnetite onto the graphene sheets will give rise to new and enhanced functionalities that show favorable magnetic properties, increased electrocatalytic activity and electron transfer ability as well as prevent the heavy aggregation [19].

Based on the above facts, we report herein a simple, cost-effective and green approach for the preparation of magnetite graphene (Fe_3_O_4_/rGO) nanocomposites by using *in-situ* one-step chemical method at room temperature. The as-prepared Fe_3_O_4_/rGO nanocomposites are used to construct modified electrodes for the simultaneous detection of DA and AA. The fabricated electrochemical sensor has exhibited rapid response, selectivity and sensitivity towards the determination of DA in the presence of AA.

## 2. Experimental Section

### 2.1. Chemicals and Reagents

Graphite flakes were purchased from Ashbury Inc. (Charlottesville, VA, USA), Sulphuric acid (H_2_SO_4_, 98%), potassium permanganate (KMnO_4_, 99.9%), hydrogen peroxide (H_2_O_2_, 30%), iron (II) sulphate (FeSO_4_·7H_2_O, 99.5%) and ammonium hydroxide (NH_4_OH, 25%) were purchased from Systerm (Shah Alam, Selangor). 3-Hydroxytyraminium chloride (dopamine, DA) and L-(+)-ascorbic acid (AA) were purchased from Merck (New York, NL, USA). Potassium hexacyanoferrate (III) (K_3_[Fe(CN)_6_]) was purchased from Sigma Aldrich (St. Louis, MO, USA). All other chemicals were of analytical grade and used without further purification. Stock solutions of DA and AA were freshly prepared using doubly-distilled (DI) water and used for electrochemical studies.

### 2.2. Characterization Techniques

Surface morphology of Fe_3_O_4_/rGO nanocomposites was examined through a FEI Nova NanoSEM 400 FESEM (Hillsboro, CA, USA) and Hitachi H-7100 (Tokyo, Japan) transmission electron microscope (TEM). Electrochemical studies such as cyclic voltammetry (CV), differential pulse voltammetry (DPV) and electrochemical impedance spectroscopy (EIS) were performed in VersaSTAT 3 electrochemical analyzer (Princeton Applied Research, Oak Ridge, TN, USA) with a conventional three-electrode system. Glassy carbon (GCE, 3 mm in diameter), Ag/AgCl and Pt wire were used as working, reference and counter electrode, respectively. Phosphate buffer solution (0.1 M) was used as a supporting electrolyte. All the electrochemical measurements were carried out at room temperature under nitrogen atmosphere.

### 2.3. Preparation of Magnetic rGO (Fe_3_O_4_/rGO) Nanocomposites

Initially, graphene oxide (GO) was synthesized from graphite using a simplified Hummers' method [20]. A GO (25 mg) was dispersed in DI water under sonication for 20 min. Then, 25% NH_4_OH solution was added dropwise into the GO solution to attain a pH value between 11 and 12. Later, the FeSO_4_ solution was slowly introduced into the GO solution under vigorous stirring at room temperature and the stirring was continued overnight. Finally, the black-coloured solution was centrifuged, washed three times with DI water for 10 min at 4000 rpm to remove the excess ammonium ions and then dried in a vacuum oven. The Fe_3_O_4_ NPs was obtained *via* the same procedure in the absence of GO and labelled as F20. Similarly, rGO was prepared through the same process without adding FeSO_4_ and labelled as G1. In the present study, Fe_3_O_4_/rGO nanocomposite was prepared by varying the weight ratios between the GO and FeSO_4_ and was denoted as G1FX, where X is the weight ratio of FeSO_4_ to GO. For the sake of convenience, four kinds of Fe_3_O_4_/rGO was prepared by fixing GO as constant with varying FeSO_4_ content (mGO: mFeSO_4_ = 1:2, 1:5, 1:10, 1:20) and they were labelled as G1F2, G1F5, G1F10 and G1F20, respectively.

### 2.4. Fabrication of Fe_3_O_4_/rGO Modified GCE

Prior to the modification, the surface of the bare GCE was polished by alumina suspension (0.05 μm) on a micro cloth polishing pad which was thoroughly rinsed with water and ultrasonically cleaned using DI water. Fe_3_O_4_/rGO (1.5 mg) was added into DI water (1 mL) and then sonicated for 1 h to get a homogeneous suspension. An aliquot (5 μL) of the suspension was drop casted on the GCE surface and dried in oven at 60 °C.

## 3. Results and Discussion

### 3.1. Characterization of Fe_3_O_4_/rGOnanocomposites

**Figure 1.**
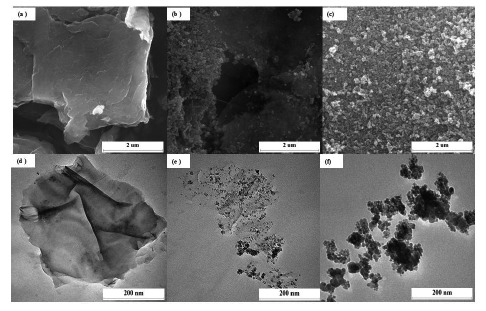
FESEM images of (**a**) G1, (**b**) Fe_3_O_4_/rGO and (**c**) F20; TEM images of (**d**) G1, (**e**) Fe_3_O_4_/rGO and (**f**) F20.

The synthesis pathway of Fe_3_O_4_/rGO nanocomposites involved the redox reaction between the GO and Fe^2+^ ions from FeSO_4_ solution. During the synthesis process, the strong oxidizing agent, GO increased the oxidation state of Fe^2+^ ions to Fe^3+^, and eventually formed into Fe_3_O_4_/rGO nanocomposites through spontaneously *in-situ* deposition of Fe_3_O_4_ nanoparticles onto the reduced graphene oxide (rGO) sheets. To investigate morphological features of the prepared G1, F20 and Fe_3_O_4_/rGO nanocomposites, FESEM and TEM images were recorded and are shown in Figure 1.

The G1 depicts a smooth surface with a wrinkled paper-like appearance (Figure 1a,d). Dispersion of Fe_3_O_4_ nanoparticles on the surface of rGO sheets has indicated a strong interaction obtained between rGO sheets and nanoparticles (Figure 1b,e). Whereas, the F20 are agglomerated that leads to larger size particles (Figure 1c,f) [21].

### 3.2. Electrochemical Behavior of Fe_3_O_4_/rGO Electrode

**Figure 2.**
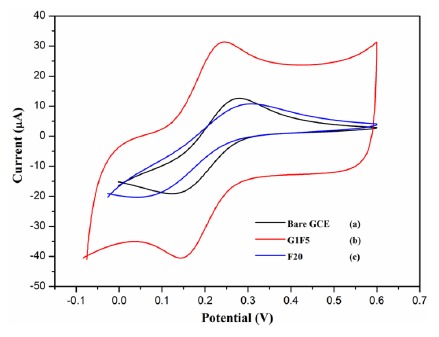
Cyclic voltammogram obtained for (**a**) bare GCE; (**b**) G1F5 and (**c**) F20 in the presence of 5 mM K_3_[Fe(CN)_6_] and 0.1 M KCl solution at a scan rate of 100 mV/s.

The electrochemical behavior of different modified electrodes was investigated to elucidate their electron transfer behavior in the presence of K_3_[Fe(CN)_6_] as a redox probe. The cyclic voltammograms for different electrodes, which are shown in Figure 2, were recorded in the presence of 5 mMK_3_[Fe(CN)_6_] and 0.1 M KCl at a scan rate of 100 mV/s. A pair of well-defined redox peak is observed with a peak to peak separation (ΔE_p_) of 140 mV for bare GCE, whereas the ΔE_p_ exhibited by the G1F5/GCE and F20/GCE is about 89 and 230 mV, respectively. When compared to the bare GCE, the redox peak current has increased significantly for the Fe_3_O_4_/rGO/GCEs. This improved electrochemical behavior can be attributed to the excellent electrical conductivity of the Fe_3_O_4_/rGO present on the electrode surface. The graphene with superior electrical conductivity and unique electron transport property could accelerate the electron transfer rate at the electrode/electrolyte interface. Only at F20/GCE, was a lower redox peak current observed than that of the bare GCE, owing to the rapid aggregation of nanoparticles that leads to larger particles (Figure 1c), which may significantly diminish the electrochemical properties of the electrode and hence be electroinactive [22–24].

**Figure 3.**
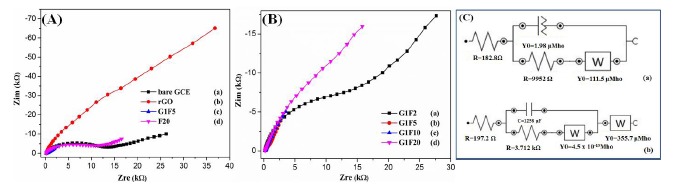
(**A**) Nyquist plots obtained for (a) bare GCE, (b) rGO, (c) G1F5 and (c) F20; (**B**) Nyquist plots for Fe_3_O_4_/rGOs (a) G1F2, (b) G1F5, (c) G1F10 and (d) G1F20 in the presence of 5 mM K_3_[Fe(CN)_6_]/K_4_[Fe(CN)_6_] (1:1) solution containing 0.1 M KCl; (**C**) Equivalent electrical circuit for (a) F20 and (b) G1F5.

Electrochemical impedance spectroscopy (EIS) is an effective tool to probe the interfacial properties of surface-modified electrodes. Figure 3 shows the Nyquist plots observed for different electrodes in the presence of 5 mM [Fe(CN)_6_]^3−/4−^ solution containing 0.1 M KCl in the frequency range from 10^−1^ to 10^4^ Hz. A Randels equivalent circuit was used in the EIS spectra to obtain value of charge transfer resistance (*R*_ct_*Ω*), where the *R*_s_, *R*_ct_, *W* and Q_CPE_ refer to the solution resistance, charge transfer resistance, Warburg impedance and constant phase element, respectively (Figure 3C) and their corresponding parameters are summarized in Table 1 [25]. The F20/GCE (Figure 3A(d)) showed a large and remarkable increase in the semicircle diameter. The high resistance (9.95 kΩ) that might cause by the aggregated Fe_3_O_4_ could diminish the electron transfer process. The rGO/GCE showed a straight line and suggests that graphene sheets can facilitate the electron transfer process. However, coupling the magnetic particles with graphene sheets, the EIS of modified electrodes display almost a straight line, indicating low interfacial electron transfer resistance and promotion of electron transfer process for the nanocomposites (Figure 3B). This may be attributed to the larger surface area and good conductivity of the Fe_3_O_4_/rGO nanocomposites [26,27]. Among the different composition of Fe_3_O_4_/rGO, the G1F5 showed very small straight line due to the high interfacial electron transfer process and low charge transfer resistant.

### 3.3. Electrocatalytic Response of Fe_3_O_4_/rGOnanocomposite Modified Electrodes towards the Oxidation of Dopamine (DA)

**Figure 4.**
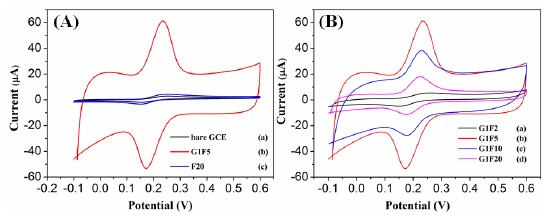
(**A**) CVs observed for (a) bare GCE, (b) G1F5 and (f) F20; (**B**) CVs for (a) G1F2, (b) G1F5, (c) G1F10 and (d) G1F20 in the presence of 0.1 M PBS (pH 6.5) containing 0.1 mM DA at a scan rate of 100 mV/s.

**Figure 5.**
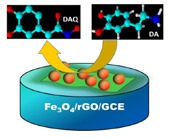
Electrocatalytic oxidation of dopamine at Fe_3_O_4_/rGO/GCE electrode.

The electrochemical behavior of different modified electrodes in the presence of 0.1 mM DA in 0.1 M PBS (pH 6.5) was investigated by cyclic voltammetric (CV) technique at a scan rate of 100 mV/s. Figure 4A shows the CV responses obtained for the bare GCE, Fe_3_O_4_/rGO/GCE and F20/GCE in the presence of 0.1 Mm DA. At a bare GCE, a well-defined redox peak was observed with the anodic and cathodic peaks at 257 mV and 148 mV, respectively. For the Fe_3_O_4_/rGO/GCEs, the oxidation peak currents were increased remarkably and the oxidation peak potentials were shifted negatively. Among the modified electrodes, the G1F5/GCE displays an enhanced electrochemical performance towards the oxidation of DA. The oxidation peak potential was negatively shifted to 234 mV with a dramatically enhanced oxidation peak current of 60.98 μA in comparison to the F20/GCE (2.87 μA) and bare GCE (2.79 μA). For comparison, electrocatalytic oxidation of DA also carried out with the Fe_3_O_4_/rGO/GCEs (Figure 4B), among them the G1F5/GCE showed higher current response than that of the other composition and these results indicate that the G1F5/GCE has high electrocatalytic activity towards the oxidation of DA. It is believed that the existence of graphene is an ideal support material and acts as an effective electron promoter for electrocatalytic oxidation of DA. Good synergistic effects emerged between Fe_3_O_4_ nanoparticles and graphene sheets enhance the conductive area and electron transfer rate between DA and electrode surface. The redox electrochemistry of DA and its oxidized form, dopaminequinone (DAQ) was studied and the proposed mechanism [28] for electrochemical behavior of dopamine is shown in Figure 5. DA is easily oxidized electrochemically and forms DAQ. When a potential is applied to the electrode, DA is easily oxidized to form DAQ after exchange of 2 electrons and 2 protons. These electrons are later donated to the electrode and produce faradaic current [29,30].

### 3.4. Effect of Scan Rate on the Electrocatalytic Oxidation of DA at G1F5/GCE Electrode

**Figure 6.**
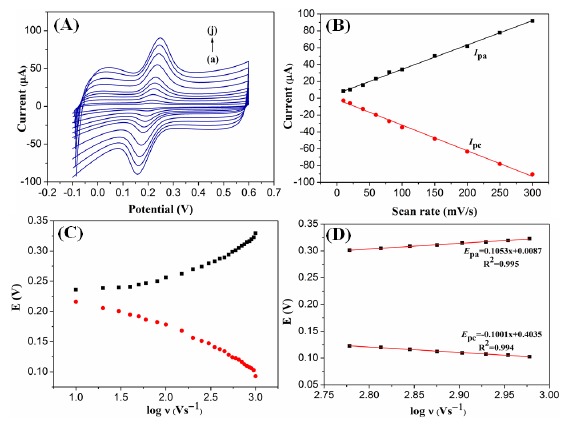
(**A**) CVs observed for G1F5/GCE in 0.1 M PBS (pH 6.5) containing 0.1 mM DA at various scan rates ((a)–(j): 10, 20, 40, 60, 80, 100, 150, 200, 250 and 300 mV/s); (**B**) The plots of peak current *versus* the scan rates; (**C**) Variation of E_p_ *versus* the logarithm of scan rate; (**D**) Variation of E_p_ *versus* the logarithm of the high scan rates: 600, 650, 700, 750, 800, 850, 900 and 950 mV/s.

The influence of scan rates on the electrocatalytic response of G1F5/GCE electrode towards DA oxidation was examined and the results are shown in Figure 6A. It can be clearly observed that the oxidation peak currents are increased linearly while the oxidation peak potential was shifted positively with scan rate in the range from 10–300 mV/s (Figure 6A).

From the CVs at modified electrode, both the values of redox peak currents (*I*_pa_ and *I*_pc_) are linearly proportional to the increasing scan rates, with linear equations of *I*_pa_ (μA) = 0.2917 + 5.144υ (mV/s) (n = 10, R = 0.9997) and *I*_pc_ (μA) = 0.3022 − 2.061υ (mV/s) (n = 10, R = 0.9997), respectively for the DA oxidation (Figure 6B). These results reveal that the electrochemical oxidation of DA at modified electrode is a typical surface adsorption-controlled process. Furthermore, a linear correlation was obtained between peak potentials (*E*_p_) and logarithm of the scan rates. For an adsorption-controlled process, the charge transfer coefficient (α) and the apparent heterogeneous electron transfer rate constant (k_s_) can be calculated from the variation of *E*_pa_ and *E*_pc_ with the logarithm of the scan rate (Figure 6C) follows the Laviron's model. Two linear regression equations of *E*_pa_ and *E*_pc_ on logν are plotted (Figure 6D) and expressed as *E*_pa_ (ν) = 0.1053 logν + 0.0087 (γ = 0.995) and *E*_pc_ (ν) = −0.1001 logν + 0.4035 (γ = 0.994), respectively. The *E*_p_ values are proportional to the logarithm of the scan rates, which is higher than 600 mV/s. The slope of the linear equation is equal to −2.3RT/ αnF and 2.3RT/(1−α)nF corresponding to the cathodic and anodic peak, respectively which can be used to evaluated the kinetic parameters, α_c_ and α_a_. R, T and F are referred to gas, temperature and Faraday constant, respectively. Here, the anodic transfer coefficient (α_a_) is found out to be 0.44. Meanwhile, the electron transfer rate constant (k_s_) is calculated to be 2.09 s^−1^ using the following Laviron's equation [31]:

$$\text{log}k_{s}=\alpha \text{log}\left(1-\alpha \right)+\left(1-\alpha \right)\text{log}\alpha -\text{log}\frac{RT}{nF\nu }-\frac{\alpha \left(1-\alpha \right)nF\Delta E_{p}}{2.3RT}$$

The calculated k_s_ value was higher than some reported values [32–34], indicating the G1F5/GCE modified electrode exhibited fast electron transfer rate towards the electrochemical oxidation of DA.

### 3.5. Effect of G1F5 Concentration on the Electrocatalytic Activity

**Figure 7.**
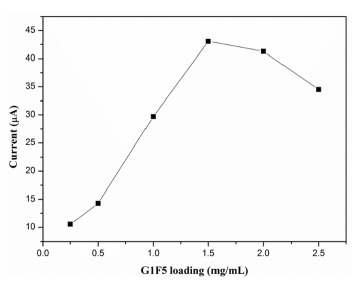
Effect of G1F5 amount on the current response of 0.1 mM DA in 0.1 M PBS (pH 6.5) at a scan rate of 100 mV/s.

The influence of G1F5 loading on the electrode surface towards the oxidation of DA was studied and the observed results are displayed in Figure 7. During the electrochemical measurement, the G1F5 volume was initially fixed as 5 μL. It is clearly seen from Figure 7 that the oxidation current response increased significantly when the concentration of G1F5 suspension increased from 0.25 mg/mL to 2.5 mg/mL due to the enhancement of the conductive surface area that influences the electron transfer rate. However, further increase of the concentration of G1F5 suspension above 1.5 mg/mL leads to a decrease in the oxidation peak current response of DA. This could be ascribed to the mass transport limitation of DA inside a thicker film and an increased mass of graphene on the electrode surface beyond the optimum level results in a shift in the oxidative over-potential towards electropositive regions and also decreases the current response [35,36]. Hence, the concentration of 1.5 mg/mL G1F5 was chosen to modify the electrode.

### 3.6. Electrochemical Detection of DA at the G1F5 Modified Electrodes

**Figure 8.**
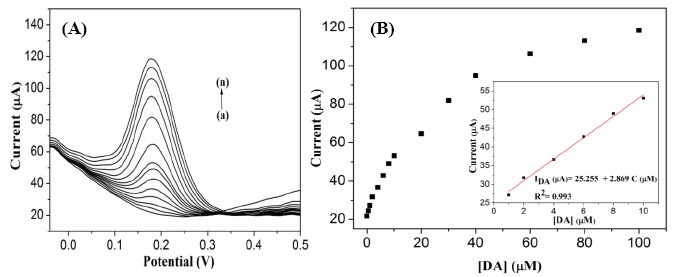
(**A**) DPV response of G1F5/GCE modified electrode with increasing the concentration of DA (from a to n: 0, 0.5, 1, 2, 4, 6, 8, 10, 20, 30, 40, 60, 80 and 100 μM). (**B**) The relationship between the oxidation peaks current against DA concentrations (0.5–100 μM). Inset: The calibration plot observed in the concentration range of 0–10 μM of DA.

The G1F5-modified GC electrode was utilized for the electrochemical detection of DA using the differential pulse voltammetry (DPV) technique. Figure 8A depicts the anodic peak current responses observed for oxidation of various concentrations of DA in the presence of G1F5/GCE. Significantly, the anodic peak current response was increases linearly with the addition of DA in the range of 0.5–100 μM (Figure 8B). The linear regression equation was expressed as *I*_DA_ (μA) = 25.255 + 2.869 C (μM) with a correlation coefficient of 0.993 (n = 14), and the detection limit (S/N = 3) was estimated to be 0.7 μM.

### 3.7. Selective Determination of DA in the Presence of AA

**Figure 9.**
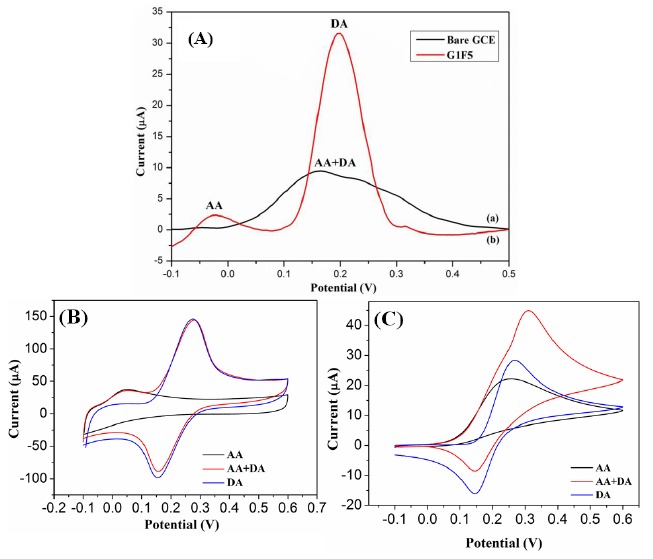
(**A**) DPV response observed for (a) bare GCE and (b) G1F5/GCE electrodes; CVs response obtained for (**B**) G1F5/GCE and (**C**) bare GCE in the presence of 0.1 mM of DA and 3 mM of AA in 0.1 M PBS (pH = 6.5).

To evaluate the selectivity of electrochemical sensor, the influence of interfering species, ascorbic acid (AA) was examined in 0.1 M PBS containing 0.1 mM DA. Figure 9A shows the DPV responses of simultaneous oxidation of DA and AA at the bare GCE and G1F5/GCE electrodes. During the simultaneous detection of DA and AA at the bare GCE, the oxidation peak potentials of AA and DA are obviously overlapping and indefinable due to the poor selectivity. AA directly interferes with DA, so the bare GCE fails to determine the individual electrochemical redox peaks for DA (Figure 9A(a)). Interestingly, the G1F5/GCE modified electrode showed a significantly increased peak current for DA as well as the overlapped peak is resolved and clearly showed the individual electrocatalytic oxidation peaks for DA and AA at about 0.19 and 0.025 V, respectively (Figure 9A(b)). In addition, the strong electrostatic interaction and π-π conjugation emerged between the aromatic regions of positively charged DA and negatively charged G1F5 makes the electron transfer favorable and enhance the oxidation of DA to electrode surface, hence the AA oxidation is inactive and inhibited [37]. Figure 9A suggests that the G1F5/GCE has excellent electrocatalytic activity towards the oxidation of DA than AA and exert no interference in the selective determination of DA. Further to confirm the selectivity of the G1F5/GCE, the cyclic voltammograms also recorded for DA, AA and DA+AA and are shown in Figure 9B and it can be clearly seen the DA and AA showed well resolved characteristic two peaks due to DA and AA. In contrast, peaks due to DA and AA are merged together in bare GCE (Figure 9C).

**Figure 10.**
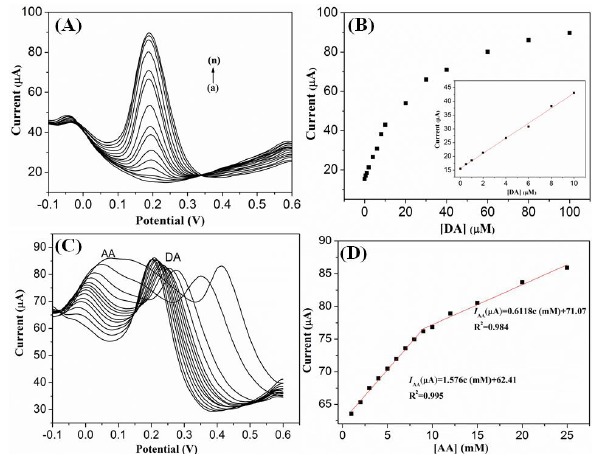
(**A**) DPV response of G1F5/GCE modified electrode with increasing the concentration of DA (from a to n: 0, 0.5, 1, 2, 4, 6, 8, 10, 20, 30, 40, 60, 80 and 100 μM) in the presence of 2 mM AA; (**B**) The relationship between the oxidation peaks current against DA concentrations (0.5–100 μM) in the presence of 2 mM AA. Inset: Enlarge view of calibration plot in the concentration range of 0–10 μM of DA; (**C**) DPVs response of G1F5/GCE modified electrode with various concentrations of AA (1–25 mM) in the presence of 0.1 mM DA; (**D**) Calibration plot obtained for oxidation peak current against AA concentrations.

The determination of DA is further investigated by differential pulse voltammetry (DPV) in the presence of AA. Figure 10A represents a series of DPV curves for the electrochemical oxidation currents for DA at a fixed concentration of 2 mM AA at G1F5/GCE. Figure 10A clearly represents that the DPV peak current for DA is linearly proportional with increasing concentration of DA in the solutions and two distinguish peaks correspond to the AA and DA are observed at about −46 and 189 mV, respectively. By varying the concentration of DA (0.5–100 μM), the anodic peak of AA is almost stable and no obvious changes in the peak current of AA are observed in the DPV curve. Hence, the coexisting species AA has induced no interference effect in the determination of DA at the G1F5/GCE.

The *I*_pa_ *versus* DA concentration curve in the range of 0–100 μM is displayed in Figure 10B. The linear regression equation was expressed as *I*_DA_ (μA) = 15.64 + 2.733 C (μM) with a correlation coefficient of 0.996 and a sensitivity of 2.733 μA/μM. The limit of detection (LOD) is estimated to be 0.12 μM (S/N = 3). Similarly, the electrochemical oxidation of AA was also investigated by varying the AA concentration while keeping the DA concentration as constant (0.1 mM) (Figure 10C). The peak current increased proportionally with increasing AA concentration. In addition, two different linear segments were observed in the calibration plots of peak currents *versus* AA concentration in the presence of 0.1 mM DA, corresponding to two different ranges of substrate concentration. The first linear segment (*I*_AA_ (μA) = 62.41 + 1.576 C (mM)) corresponds to AA concentration from 1–9 mM, while the second linear segment (*I*_AA_ (μA) = 71.07 + 0.6118 C (mM)) corresponds to AA concentration from 9–25 mM (Figure 10D). The decrease in sensitivity (slope) at higher linear range of AA is attributed to the kinetic limitation of the G1F5 [38]. The detection limit for AA in the lower range and higher range are found out to be 0.42 μM and 2.77 μM, respectively. Furthermore, shift in the peak potential of AA is likely due to the acidic nature of AA that affects and changes the pH of the solution when excess AA was added [39,40]. Compared with other works (Table 2), it is noteworthy to mention that the modified electrode based on Fe_3_O_4_/rGO nanocomposite greatly increases the electrocatalytic active sites and promotes the electron transfer in the detection of DA. Furthermore, the reproducibility of the G1F5/GCE was also evaluated by the repetitive electrochemical measurements in the solution containing 0.1 mM DA. The modified electrode gives a relative standard deviation (R.S.D) of around 3.0% after 10 successive measurements, implying good reproducibility of the modified electrode.

## 4. Conclusions

In conclusion, Fe_3_O_4_/rGO nanocomposites were successfully synthesized *via* a simple, cost-effective and green approach. A sensitive and selective electrochemical sensor with Fe_3_O_4_/rGO nanocomposites was developed for the determination of DA in the presence of AA. The Fe_3_O_4_/rGO nanocomposites modified electrode showed significantly improved peak current towards the oxidation of DA. The Fe_3_O_4_/rGO based electrochemical sensor displayed excellent electrocatalytic activity and electron transfer rate towards the DA oxidation. Moreover, the sensor electrode showed good sensitivity, selectivity and low detection limit in the simultaneous detection of DA and AA. These results reveal that Fe_3_O_4_/rGO/GCE could be a potential candidate for electrochemical and biosensor applications.

## Figures and Tables

**Table 1.** EIS fitting parameters for modified electrodes.

**Electrochemical Impedance Fitting Parameters**				
Electrodes	***R_s_* (Ω)**	***Q_CPE_* (*F*)**	***R_ct_* (Ω)**	***W***
**G1F5**	197.2	1.26 × 10^−3^	3.7 × 10^−3^	3.56 × 10^−4^
**F20**	182.8	1.98 × 10^−6^	9952	1.11 × 10^−4^

*Footnotes*: R_s_—Solution resistance; Q_CPE_—constant phase element; R_ct_—Charge transfer resistance; W—Warburg impedance.

**Table 2.** Comparison of results for the determination of DA using different electrodes.

**Electrode**	**Method**	**Interference**	**Linearity (μM)**	**Detection Limit (μM)**	**Ref.**
CAT ^a^/ZnONps/CPE ^b^	CA, CPA	-	5–41	3	[41]
Nanostructured gold	DPV	AA	10–100	5	[42]
Cu_2_O/GCE	CV	UA	0.1–10	0.01	[43]
TiO_2_–graphene/GCE	CV	UA, AA	5–200	2	[44]
2-Amino-thiazol (AT) film/GCE	DPV	UA	5–25	5	[45]
Graphene/GCE	CV	EP, UA, AA	2.5–100	0.5	[46]
CTAB ^c^/GNSP ^d^	DPV	AA	4–52	0.6	[47]
Meso-SiO_2_/CPE	DPV	UA, AA	0.4–25	0.1	[48]
RGO-HDPPy ^e^ doped GCE	DPV	UA, AA	0.001–8	0.003	[49]
Fe_3_O_4_/rGO/GCE	DPV	AA	0.5–100	0.12	Present work

*Footnotes*:

a Catalase;

b Carbon Paste Electrode;

c Cetyltrimethylammonium bromide;

d Graphene nano-sheets;

e Polypyrrole nanospheres with highly dispersibility.
